# Overexpression of CDT1 inhibits cell cycle progression at S phase by interacting with the mini‐chromosome maintenance complex and causes DNA damage

**DOI:** 10.1002/2211-5463.70127

**Published:** 2025-09-26

**Authors:** Takashi Tsuyama, Nonoka Takayama, Rina Tanaka, Yuuki Arai, Yohko Yamaguchi, Yuko Nawata, Yutaro Azuma, Shusuke Tada

**Affiliations:** ^1^ Department of Molecular Biology, Faculty of Pharmaceutical Sciences Toho University Chiba Japan; ^2^ Department of Molecular Toxicology, Faculty of Pharmaceutical Sciences Toho University Chiba Japan

**Keywords:** CDT1, CMG helicase, DNA replication licensing, replication fork, replication stress

## Abstract

Cdc10‐dependent transcript 1 (CDT1) is an essential protein for DNA replication licensing, which loads the mini‐chromosome maintenance (MCM) complex onto replication origins. We previously reported that excess CDT1 inhibits the elongation of nascent strands during DNA replication in *Xenopus* egg extracts. In the present study, we investigated the underlying mechanism through which CDT1 inhibits replication fork progression by expressing various CDT1 mutants in human cells. Initiation of DNA replication resulted in downregulation of CDT1, preventing MCM reloading within the same cell cycle; thus, CDT1 overexpression induces rereplication. In this study, we observed that overexpression of a mutant CDT1 lacking licensing activity induced cell cycle arrest at the S phase in human cells. An additional mutation in the MCM‐binding domain reduced this cell cycle inhibitory effect. Furthermore, overexpression of CDT1 induced DNA damage independent of its licensing activity. These results suggest that CDT1 overexpression inhibits the progression of replication forks by interacting with the MCM complex, leading to the stalling and collapse of replication forks.

## Graphical abstract

CDT1 is an essential protein for DNA replication licensing that loads the MCM complex, the eukaryotic replicative DNA helicase, onto replication origins. Overexpression of CDT1 induces cell cycle arrest at the S phase. Here we showed CDT1 inhibits the progression of replication forks by interacting with the MCM complex, leading to the stalling and collapse of replication forks.

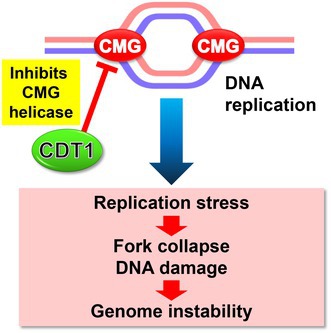

**Abbreviations**
ATR: ataxia telangiectasia and Rad3‐related
DSB: DNA double‐strand break
GST: glutathione S‐transferase
pre‐RC: pre‐replicative complex
WHD: winged‐helix domain

To maintain genomic integrity, DNA must be precisely duplicated during the cell division cycle, and inaccurate DNA duplication triggers chromosomal aberrations, which can lead to tumor formation [1]. The initiation of DNA replication is strictly regulated by the DNA replication licensing system, which ensures that DNA replication occurs only once per cell cycle. In the late M to early G1 phase, origin recognition complex (ORC), cell division cycle 6 (CDC6), and Cdc10‐dependent transcript 1 (CDT1) bind to replication origins in a stepwise manner to recruit mini‐chromosome maintenance (MCM) complex composed of Mcm2‐7 proteins to form a pre‐replicative complex (pre‐RC) [2]. During the S phase, the MCM complex in the pre‐RC undergoes conversion into the Cdc45‐MCM‐GINS complex (CMG helicase), an active form of the replicative DNA helicase in eukaryotes. Activating replicative DNA helicases requires the concerted action of Dbf4‐dependent kinase (DDK) and cyclin‐dependent kinase. Pre‐RC formation is suppressed during the S, G2, and M phases to prevent the re‐initiation of DNA synthesis at origins that have already fired. This regulation is crucial because the duplication of an already replicated chromosome region (rereplication) can lead to DNA damage and genome instability.

CDT1 is a major target for the regulation of licensing activities in higher eukaryotes. Upon initiation of the S phase, CDT1 undergoes inactivation via multiple mechanisms, including the binding of geminin, an endogenous inhibitor, and degradation via ubiquitin‐dependent proteolysis [3]. CDT1 was shown to directly bind to Mcm6 through its C‐terminal domain, which is essential for origin licensing [4, 5]. Conversely, geminin binds to CDT1 and prevents its interaction with the MCM complex [6]. In addition, two E3 ubiquitin ligases, CRL4^CDT2^ and SCF^SKP2^, were found to be redundantly involved in the degradation of CDT1 in human cells. CDT1 binds to proliferating cell nuclear antigen (PCNA) through its N‐terminal PCNA‐interacting protein box (PIP‐box) after PCNA is loaded onto chromatin in the S phase, triggering CRL4^CDT2^‐mediated CDT1 degradation [7, 8, 9, 10]. In human cells, CDT1 undergoes CDK2‐mediated phosphorylation in the S phase, modulated by its interaction with cyclin A via the cyclin‐binding motif (Cy‐motif). This phosphorylation provides a binding site for SCF^SKP2^ ubiquitin ligase, leading to the ubiquitination and degradation of CDT1 [11, 12]. CDT1 is regulated by APC/C^CDH1^, a ubiquitin ligase that recognizes the destruction box (D‐box) of its substrate. Thus, a mutation in the D‐box of CDT1 induces remarkable rereplication [13].

Elevated levels of CDT1 have been observed in numerous tumor cells and were found to be associated with poor prognosis [14, 15, 16, 17, 18]. In certain cancer cell lines or transformed cells, forced overexpression of CDT1 induces rereplication [19, 20]. Replication stress caused by obstacles that retard or arrest DNA replication is a driver of genomic instability and a hallmark of cancer cells [21]. Thus, the rereplication induced by CDT1 overexpression could be a major source of replication stress in cancer cells. Reportedly, rereplication leads to the accumulation of single‐strand DNA (ssDNA) and the formation of DNA double‐strand breaks (DSBs), activating ataxia telangiectasia and Rad3‐related (ATR) and ataxia telangiectasia‐mutated checkpoint pathways, respectively [19, 22, 23]. Among these checkpoint kinases, ATR has crucial pleiotropic functions in the replication stress response and phosphorylates multiple downstream substrates, including the effector kinase CHK1. CHK1 suppresses replication‐origin firing, arrests cell cycle progression, and stabilizes stalled replication forks.

We previously reported that excess CDT1 inhibits the elongation of nascent strands during DNA replication in *Xenopus* egg extracts, independent of rereplication or checkpoint activation [24]. Unlike the uncoupling of parent DNA and nascent DNA synthesis, which occurs upon inhibition of DNA polymerase [25], the CDT1‐mediated inhibition of DNA synthesis was not coupled with increased ssDNA exposure. These results suggest that CDT1 inhibits the progression of CMG helicase rather than DNA polymerase activity [26]. Given that replication inhibition by ectopic CDT1 may lead to genome instability, potentially resulting in tumorigenesis or tumor progression, it is crucial to elucidate the molecular mechanisms through which CDT1 inhibits replication fork progression. In the present study, we investigated the underlying mechanism through which CDT1 inhibits replication fork progression by expressing various CDT1 mutants in human cells. Herein, we found that CDT1 overexpression inhibited S‐phase progression, regardless of its licensing activity. This inhibition was abolished by mutating the MCM‐binding domain, suggesting that CDT1 inhibits replication fork progression by directly interacting with the MCM complex. Moreover, overexpression of mutant CDT1, in which the licensing activity was attenuated, induced DNA DSB formation. These results suggest that the overexpression of CDT1 induces not only rereplication but also the stall of replication forks by inhibiting CMG helicase activity, followed by the generation of DNA damage that leads to genome instability.

## Materials and methods

### Cell culture, cell lines, and genome editing

MCF‐7 (RRIR: CVCL_0031) (JCRB0134, Japanese Collection of Research Bioresources [JCRB] cell bank) cells were cultured in DMEM supplemented with 10% fetal bovine serum at 37 °C in a humid atmosphere containing 5% CO_2_. Cell lines ectopically expressing wild‐type CDT1 or mutant CDT1 in a doxycycline‐inducible manner were generated through CRISPR‐Cas9‐based insertion of expression constructs into the genomic DNA of MCF‐7 cells. Human CDT1 cDNA and DNA encoding a FLAG‐tag were cloned into the pTetOne vector (Takara Bio Inc., Shiga, Japan) to express FLAG‐tag‐fused CDT1. To construct a plasmid expressing the mutant CDT1, the DNA sequence encoding the nuclear localization signal (NLS) of the SV40 T antigen was cloned into the pTetOne vector along with the mutant CDT1 cDNA. PCR was performed to linearize plasmid DNA using the following primers: forward primer: 5′‐CTTTCACCAGCGTTTCTGGGTGAGC‐3′ and reverse primer: 5′‐GGTTTCGCCACCTCTGACTTGAGCG‐3′. The linearized DNA was transfected using Lipofectamine 3000 Transfection Reagent (Thermo Fisher Scientific, Waltham, MA, USA) with linearized puromycin‐resistant gene and pCas9 (BB)‐2A‐GFP (pX458) plasmid containing adeno‐associated virus integration site 1 (AAVS1) locus targeting guide RNA sequence (T1 target sequence [27]); 5′‐GTCCCCTCCACCCCACAGTG‐3′. Colonies resistant to 0.5 μg·mL^−1^ of puromycin were selected, and the expression of the inserted gene in the presence of 1 μg·mL^−1^ of doxycycline was confirmed by RT‐PCR and immunoblotting. All experiments were conducted using cells confirmed to be free of mycoplasma contamination.

### Gene transduction

For transient expression, cDNA encoding mutant CDT1 was inserted into the pCMV‐3Tag‐6 vector (Agilent Technologies, Santa Clara, CA, USA) adjacent to DNA sequences encoding the 3x Myc tag and NLS, followed by transfection into MCF‐7 cells using the Neon NxT Electroporation System (Thermo Fisher Scientific) by applying an electric pulse of 1100 V, 30 ms, twice.

### Recombinant proteins and antibodies

Complementary DNA sequences of recombinant proteins were inserted into the pGEX‐6P‐3 vector (GE Healthcare, Chicago, IL, USA) with a FLAG‐tag sequence. Recombinant proteins were expressed in BL21 (DE3)‐CodonPlus‐RIL or BL21 (DE3) pLysS competent cells (Agilent Technologies) and purified using Glutathione Sepharose 4 B (GE Healthcare). Anti‐FLAG M2 monoclonal antibody was purchased from Sigma‐Aldrich (St. Louis, MO, USA). Anti‐DYKDDDDDK monoclonal antibody (clone no. 2H8) was purchased from Medical Chemistry Pharmaceutical (Kobe, Japan). Anti‐human Mcm6 (13347‐2‐AP), anti‐human RPA2 (10412‐1‐AP), anti‐human GAPDH (10494‐1‐AP), anti‐Chk1 (25887‐1‐AP), anti‐phospho‐Chk1 (Ser317) polyclonal antibody (28807‐1‐AP), and anti‐human Lamin B1 (12987‐1‐AP) polyclonal antibodies were purchased from Proteintech (Rosemont, IL, USA). Anti‐human MRE11 polyclonal antibody (#4895), anti‐phospho‐ATR (Thr1989) rabbit monoclonal antibody (D5K8W) (#30632), and anti‐Myc‐tagged mouse monoclonal antibody (9B11) (#2276) were purchased from Cell Signaling Technology (Danvers, MA, USA). Anti‐human RAD51 polyclonal antibody (GTX100496) and anti‐ATR antibody (GTX128146) were purchased from GeneTex (Irvine, CA, USA). Anti‐phospho‐histone H2A.X (Ser139) monoclonal antibody (JBW301) (05‐636‐l) was purchased from Merck Millipore (Billerica, MA, USA).

### GST pull‐down assay

MCF‐7 cells were lysed by sonication in lysis buffer (40 mm HEPES‐KOH [pH 7.6], 50 mm KCl, 0.1% Triton X‐100, 2 mm MgCl_2_) supplemented with a protease inhibitor cocktail (Nacalai Tesque, Kyoto, Japan) and 0.5 mm phenylmethylsulfonyl fluoride (Tocris Bioscience, Bristol, UK). After centrifugation for 10 min at 16 600 ***g***, the supernatant was collected, and the protein concentration was quantified using the TaKaRa BCA Protein Assay Kit (Takara Bio Inc., Shiga, Japan). GST‐fused wild‐type or mutant CDT1 protein (100 nm) adsorbed onto Glutathione Sepharose 4 B was incubated with 150 μg of MCF‐7 cell lysate supplemented with 25 units·mL^−1^ of Benzonase^®^ Nuclease (Merck Millipore) for 120 min at 4 °C. After incubation, the beads were washed three times with 200 μL of lysis buffer, and the proteins bound to the beads were eluted with 20 μL elution buffer composed of 40 mm HEPES‐KOH [pH 7.6], 50 mm KCl, 0.1% Triton X‐100, 2 mm MgCl_2_, and 40 mm glutathione. Proteins in the reaction mixture (i.e., input proteins) and proteins eluted from glutathione Sepharose beads were detected by immunoblotting.

### Detection of ATR and CHK1 phosphorylation

In brief, 3.5 × 10^5^ of CDT1‐NGB‐NDL expressing cells were lysed in 90 μL of Sample Buffer Solution with Reducing Reagent (6×) for sodium dodecyl sulfate‐polyacrylamide gel electrophoresis (Nacalai Tesque, Kyoto, Japan), diluted with phosphate‐buffered saline (PBS), supplemented with 25 U Benzonase^®^ (Merck Millipore) and 1 mm CaCl_2_. The cell lysates were incubated for 30 min at room temperature, followed by heating at 96 °C for 3 min. Proteins were separated using a 5–20% polyacrylamide gel (EHR‐R520L; Atto, Osaka, Japan) and EzRun MOPS buffer (WSE‐7065; Atto) and blotted onto polyvinylidene fluoride membranes. The membrane was blocked with Blocking One (Nacalai Tesque) for 1 h at room temperature and incubated overnight with a primary antibody diluted in Solution 1 of Can Get Signal™ Immunoreaction Enhancer Solution (TOYOBO, Osaka, Japan) at 4 °C. After washing with Tris‐buffered saline containing 0.05% Tween 20 (TBS‐T), the membrane was incubated with a horseradish peroxidase‐conjugated secondary antibody diluted in Solution 2 of Can Get Signal™ Immunoreaction Enhancer Solution (TOYOBO) for 1 h at room temperature, followed by washing with TBS‐T again. ImmunoStar LD (FUJIFILM Wako Chemicals, Osaka, Japan) was used for the chemiluminescent detection of immunoblot signals.

### Flow cytometry

Cells were harvested and fixed with ice‐cold 70% ethanol overnight at −20 °C. After washing with PBS containing 0.05% Tween 20 (PBS‐T), cells were incubated with PBS‐T supplemented with 20 μg·mL^−1^ propidium iodide, 4 μg·mL^−1^ RNase A, and 10 U·mL^−1^ RNase T_1_ at 37 °C for 30 min. The cells were subjected to FACSCalibur (BD Bioscience, San Jose, CA, USA), and the results were analyzed using CellQuest software. For detection of 5‐ethynyl‐2′‐deoxyuridine (EdU) incorporation, cells were pulse‐labeled for 2 h with 10 μm EdU. Samples were processed for flow cytometry using the Click‐iT EdU Alexa Fluor 488 Flow Cytometry Assay Kit (Thermo Fisher Scientific, MA, USA), and DNA was counterstained with 1 μg·mL^−1^ of 7‐aminoactinomycin D (Fujifilm Wako Pure Chemicals Corp., Osaka, Japan).

### Cell fractionation

Cells (5 × 10^5^) were resuspended in 50 μL of buffer A (10 mm HEPES [pH 7.6], 10 mm KCl, 1.5 mm MgCl_2_, 0.34 m sucrose, 10% glycerol, 1 mm DTT, supplemented with protease inhibitor cocktail) containing 0.1% Triton X‐100 and incubated for 5 min on ice. After centrifugation at 1300 ***g*** for 5 min at 4 °C, the nuclei were collected from pellet 1 (P1). The supernatant was clarified by further centrifugation at 18 000 ***g*** for 15 min at 4 °C and collected as the soluble fraction. P1 was washed once with buffer A and then incubated in 100 μL of solution B (3 mm EDTA, 0.2 mm EGTA, 1 mm DTT, supplemented with protease inhibitor cocktail) for 5 min on ice. The chromatin fraction was collected as the precipitate after centrifugation at 1700 ***g*** for 5 min at 4 °C and washed once with buffer B. The fraction was resuspended in 50 μL 2× Laemmli SDS sample buffer. Proteins in 10 μL of the soluble and chromatin fractions were detected by immunoblotting. For analyzing aphidicolin‐treated cells, 20 μm aphidicolin was added to the cells 30 h prior to collection, and proteins in 20 μL of the soluble and chromatin fractions were detected by immunoblotting.

### DNA‐binding assay

GST‐ and FLAG‐tagged wild‐type or mutant CDT1 proteins (200 nm) were incubated with double‐stranded or single‐stranded DNA cellulose (dsDNA cellulose or ssDNA cellulose; Sigma‐Aldrich, MO, USA) in binding buffer (20 mm Tris/HCl [pH 7.5], 50 mm NaCl, and 0.1% Triton X‐100) for 1 h at 4 °C. Following incubation, the beads were washed with binding buffer, and the bound proteins were eluted with 2× Laemmli SDS sample buffer. Proteins in the eluted fractions were analyzed by immunoblotting.

### Cell viability test

Cell viability was measured using the CellTiter‐Glo® Luminescent Cell Viability Assay Kit (Promega, Madison, WI, USA) according to the manufacturer's instructions. Briefly, 3000 cells were seeded in a 96‐well plate and incubated with or without 1 μg·mL^−1^ doxycycline at 37 °C. After incubation, the CellTiter‐Glo reagent was added to each well and mixed for 2 min using an orbital shaker. Subsequently, the mixtures were incubated at room temperature for 10 min, and the luminescence intensity was measured using a DTX800 Multimode Detector (Beckman Coulter, Brea, CA, USA) and expressed as arbitrary units.

### Data collection and presentation

All experiments were repeated at least three times, and representative data are presented.

## Results

### CDT1 inhibits replication fork progression in a manner independent of its licensing activity

**Fig. 1:**
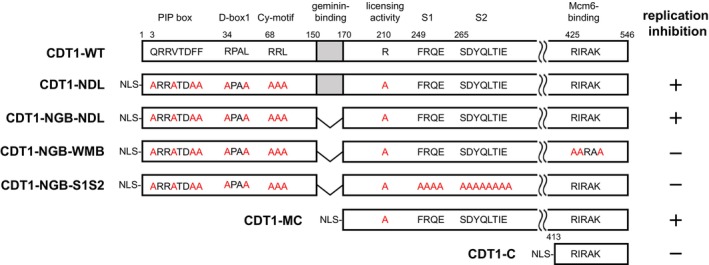
Schematic illustration of CDT1 mutants and their ability to associate with Mcm6. Schematic representation of CDT1 mutants used in this study. Results of their ability to inhibit replication are summarized on the right. Mutated amino acid residues are highlighted in red.

**Fig. 2:**
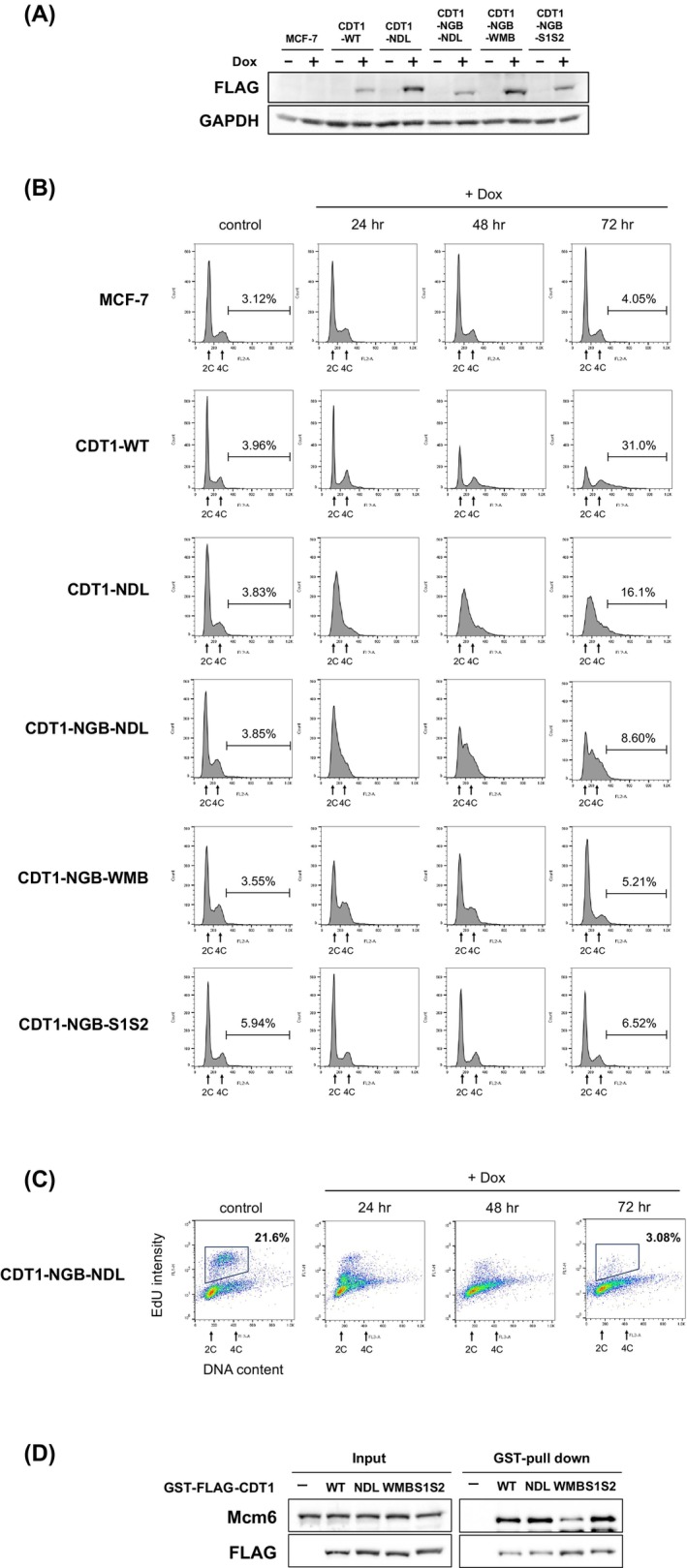
Inhibition of cell cycle progression by overexpression of CDT1. (A) Wild‐type CDT1, CDT1‐NDL, CDT1‐NGB‐NDL, CDT1‐NGB‐WMB, or CDT1‐NGB‐S1S2 fused with FLAG‐tag was expressed in recombinant MCF‐7 cells by incubating with 1 μg·mL^−1^ of doxycycline (Dox) for 72 h. Cell lysate containing 100 μg of total protein was subjected to sodium dodecyl sulfate–polyacrylamide gel electrophoresis and analyzed by immunoblotting using the indicated antibodies (*n* = 3). (B, C) Cell cycle profiles of wild‐type CDT1 or CDT1 mutant‐expressing cells in single‐parameter flow cytometry (B) or CDT1‐NGB‐NDL‐expressing cells in two‐dimensional flow cytometry (C) at 24, 48, and 72 h after the addition of 1 μg·mL^−1^ of doxycycline (*n* = 3). (D) Glutathione Sepharose was incubated with lysates from MCF‐7 cells supplemented with purified GST‐ and FLAG‐tag fused recombinant CDT1 mutant proteins lacking the geminin‐binding domain. Mcm6 that was adsorbed on the beads was detected by immunoblotting (*n* = 3).

To clarify the mechanism through which CDT1 inhibits replication fork progression, we constructed a CDT1 expression plasmid carrying a reverse tetracycline transactivator and a tetracycline response element in the promoter for ectopic expression of CDT1. This construct was inserted into the genome of MCF‐7 cells to establish the doxycycline‐inducible CDT1 expression cell lines. We also established cell lines expressing a mutant form of CDT1 that possesses reduced licensing activity by substituting arginine 210 with alanine [28], disrupting the PIP‐box, Cy‐motif, and D‐box to prevent degradation, with the NLS of the SV40 T antigen fused to the N terminus. Herein, the mutant CDT1 is referred to as CDT1‐NDL, which is a nondegradable mutant with attenuated licensing activity (Fig. 1). The established cell lines expressed ectopic CDT1 in a doxycycline‐dependent manner (Fig. 2A). Flow cytometry was performed to assess cell cycle progression in cells ectopically expressing wild‐type CDT1 (CDT1‐WT) or CDT1‐NDL (Fig. 2B). Induction of wild‐type CDT1 markedly increased the proportion of cells with greater than 4C DNA presumably due to rereplication. In contrast, the expression of CDT1‐NDL tended to arrest the cell cycle in the S phase, although substantial numbers of re‐replicating cells were also observed. We speculated that excess CDT1‐NDL could sequester geminin from endogenous CDT1, thereby inducing rereplication, as indicated previously [29]. We then generated an additional cell line to express a mutant protein lacking the ability to bind geminin by deleting part of the geminin‐binding domain (150–170 amino acid region) from CDT1‐NDL (non‐geminin‐binding CDT1‐NDL: CDT1‐NGB‐NDL; Fig. 1) [30]. Ectopic expression of CDT1‐NGB‐NDL enhanced the proportion of cells in the S phase while markedly reducing the over‐4C population (Fig. 2A,B). Furthermore, two‐dimensional flow cytometry analysis revealed that the expression of CDT1‐NGB‐NDL resulted in a marked decrease in EdU incorporation into cells with DNA content of between 2C and 4C (Fig. 2C). These findings suggested that CDT1‐NGB‐NDL inhibits the progression of replication forks in human cells without inducing rereplication, which is consistent with our previous results obtained using *Xenopus* egg extracts [24, 26].

### The ability to interact with the MCM complex is indispensable for CDT1 to inhibit replication fork progression

Our previous report suggests that CDT1 inhibits nascent strand elongation by repressing the progression of the replication fork in *Xenopus* egg extract [24, 26]. Recently, CDT1 was shown to directly inhibit the progression of the CMG helicase at the replication fork, limiting DNA synthesis until CDT1 undergoes degradation during the normal cell cycle [31]. Hence, we examined whether the interaction of CDT1 with the MCM complex was necessary for cell cycle arrest in the S phase. Given that arginine 425, isoleucine 426, and lysine 429 residues in human CDT1 are known to be involved in the interaction with Mcm6 [32], these amino acids of CDT1‐NGB‐NDL were substituted with alanine (weak MCM‐binding CDT1‐NGB‐NDL: CDT1‐NGB‐WMB in Fig. 1). We confirmed that CDT1‐NGB‐WMB showed a decreased affinity for Mcm6, whereas CDT1‐NGB‐NDL showed an affinity comparable to that of wild‐type CDT1 (Fig. 2D). Next, we established a doxycycline‐inducible CDT1‐NGB‐WMB expression cell line and examined whether the expression of CDT1‐NGB‐WMB could induce cell cycle arrest in the S phase. The results revealed that the expression of CDT1‐NGB‐WMB had no significant effect on cell cycle progression (Fig. 2A,B), suggesting that the association of CDT1 with Mcm6 plays a role in inhibiting replication fork progression.

### The middle region of CDT1 is required for inhibiting replication fork progression

**Fig. 3:**
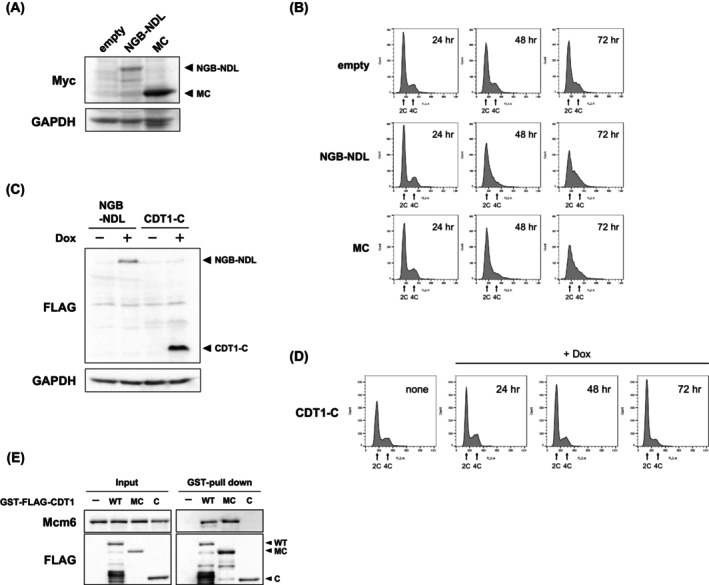
Expression of CDT1‐MC, but not CDT1‐C, inhibits cell cycle progression at the S phase. (A, B) MCF‐7 cells were transfected with pCMV‐3Tag‐6 (empty), CDT1‐NGB‐NDL/pCMV‐3Tag‐6 (NGB‐NDL), or CDT1‐MC/pCMV‐3Tag‐6 (MC) to express Myc‐tagged CDT1 mutant proteins. Cells were collected after incubation for 24, 48, and 72 h (*n* = 3). (A) After a 72‐h incubation, proteins in the cells were detected by immunoblotting using the indicated antibodies. (B) The cell cycle profiles of collected cells were analyzed by flow cytometry. (C, D) Expression of FLAG‐tagged CDT1‐NGB‐NDL or CDT1‐C was induced by incubation of transfected cells with 1 μg·mL^−1^ of doxycycline for 24, 48, and 72 h (*n* = 3). (C) After a 72‐h incubation with doxycycline, proteins in the cells were detected by immunoblotting using the indicated antibodies. (D) The cell cycle profiles of collected cells were analyzed by flow cytometry. (E) Glutathione Sepharose was incubated with the lysate of MCF‐7 cells ectopically expressing GST‐ and FLAG‐tag fused recombinant CDT1 mutant proteins. After incubation, Mcm6 adsorbed on the beads was detected by immunoblotting (*n* = 3).

The CDT1 structure is divided into three functional regions: the N‐terminal, middle, and C‐terminal regions [3, 5]. CDT1 contains two tandem winged‐helix domains (WHD): one in the middle and the other in the C‐terminal region [6, 33]. The C‐terminal WHD interacts with Mcm6, whereas the role of WHD in the middle region remains elusive. To examine whether WHD in the middle region plays a role in inhibiting replication forks, deletion mutants of CDT1 comprising the middle and C‐terminal regions (CDT1‐MC) or only the C‐terminal region (CDT1‐C) were expressed in MCF‐7 cells, and their effects on cell cycle progression were analyzed (Fig. 3). As we could not establish a stable CDT1‐MC‐expressing cell line, we introduced CDT1‐NGB‐NDL or CDT1‐MC expression plasmids into MCF7 cells and examined the effect of transient expression of the mutant proteins on the cell cycle profile (Fig. 3A). The transfected cells gradually accumulated in the early to mid‐S phase from 48‐ to 72‐h post‐transfection with the CDT1‐NGB‐NDL or CDT1‐MC expression plasmid (Fig. 3B). In contrast, doxycycline‐induced expression of CDT1‐C exerted a minimal impact on cell cycle progression (Fig. 3C,D). The GST pull‐down assay showed that CDT1‐MC, but not CDT1‐C, interacted efficiently with Mcm6 in the cell lysate (Fig. 3E), suggesting that the middle region is also responsible for the interaction with Mcm6.

We recently reported that CDT1 self‐associates with WHD in the middle region [34]. A mutation in two β‐strands (S1, S2) of the domain did not affect the interaction with other pre‐RC components, including Orc2, CDC6, Mcm2, and Mcm6, although it reduced the self‐association of CDT1. Next, we examined whether the mutation in the two β‐strands attenuates the ability of CDT1 to arrest the cell cycle in the S phase. Four amino acids in S1 and seven amino acids in S2 of CDT1‐NGB‐NDL were substituted with alanine (CDT1‐NGB‐S1S2) (Fig. 1). Although CDT1‐NGB‐S1S2 bound Mcm6 to an extent comparable to that of wild‐type CDT1 (Fig. 2D), the expression of CDT1‐NGB‐S1S2 had a limited impact on cell cycle progression (Fig. 2A,B). These results suggested that WHD in the middle region, in addition to the MCM complex‐binding property in the C‐terminal region, is required for CDT1 to inhibit replication fork progression.

These results suggested that the C‐terminal WHD of CDT1 is crucial, although insufficient for interaction with the MCM complex, and that the middle WHD contributes to the inhibition of replication fork progression.

### CDT1 mutants, which lack the ability to inhibit DNA replication, showed reduced affinity for chromatin

**Fig. 4:**
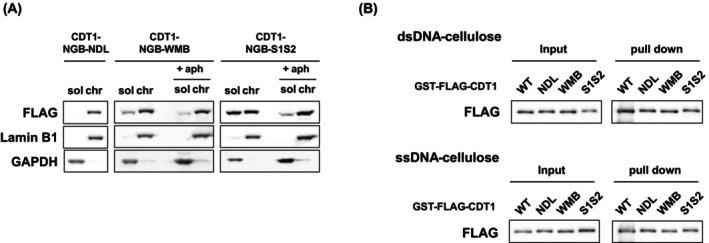
Chromatin binding of CDT1 mutants in human cells. (A) FLAG‐tagged CDT1 mutant‐expressing cells were incubated with 1 μg·mL^−1^ doxycycline for 72 h for CDT1‐NGB‐NDL, CDT1‐NGB‐WMB, or CDT1‐NGB‐S1S2 expressing cells in the absence or presence of aphidicolin (+ aph). After incubation, cells were fractionated into detergent‐soluble (sol) and detergent‐insoluble chromatin (chr) fractions. The proteins in each fraction were detected by immunoblotting using the indicated antibodies (*n* = 3). (B) Double‐stranded or single‐stranded DNA cellulose was incubated with purified GST‐ and FLAG‐tag fused recombinant CDT1 mutant proteins lacking the geminin‐binding domain. After incubation, bound proteins were detected by immunoblotting (*n* = 3).

To determine whether CDT1‐NGB‐NDL inhibits cell cycle progression by binding to MCM helicase on replicating chromatin, chromatin binding of CDT1 mutants was examined. Upon inducing CDT1 mutant expression, cell lysates were fractionated into detergent‐soluble and ‐insoluble (chromatin) fractions (Fig. 4A). CDT1‐NGB‐NDL, which suppresses S phase progression, was detected exclusively in the chromatin fraction. Conversely, CDT1‐NGB‐WMB and CDT1‐NGB‐S1S2, both of which did not affect the cell cycle, were detected in the soluble and chromatin fractions. To examine whether the difference is due to the affinity of CDT1 for DNA, we assessed the influences of the mutations on the DNA‐binding ability of CDT1 (Fig. 4B). The mutations did not affect the affinity for either double‐stranded or single‐stranded DNA (Fig. 4B), suggesting that CDT1‐NGB‐NDL inhibits replication fork progression through its association with proteins on the chromatin, rather than by binding to DNA.

To eliminate the possibility that the difference was due to the population of S‐phase cells, chromatin binding of CDT1‐NGB‐WMB and CDT1‐NGB‐S1S2 was examined in the presence of aphidicolin to stall the cell cycle at the S phase. However, similar results were observed under this condition. These results indicated that CDT1 inhibits DNA replication by associating with chromatin; however, chromatin binding of CDT1‐NGB‐WMB and CDT1‐NGB‐S1S2 is insufficient to prevent replication fork progression. Both the WHD in the middle region and the C‐terminal MCM‐binding domain may be essential for the binding of CDT1 to CMG helicase at the replication fork to efficiently suppress its function.

### Genome instability induced by ectopic CDT1 expression is not due to rereplication

**Fig. 5:**
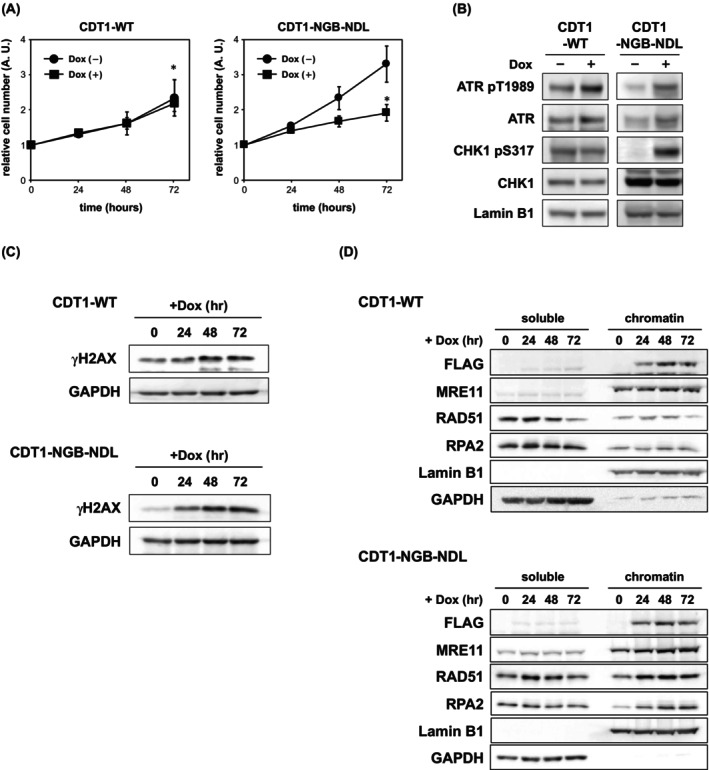
Ectopic expression of CDT1‐NGB‐NDL induces DNA damage. (A) Numbers of viable cells were analyzed until 72‐h incubation with or without induction of wild‐type CDT1 (CDT1‐WT) or CDT1‐NGB‐NDL expression by adding 1 μg·mL^−1^ of doxycycline. Error bar indicates SEM. *P < 0.05 (one‐sided Student's *t*‐test, *n* = 3) (B) CDT1‐WT or CDT1‐NGB‐NDL expressing cells were collected after 24‐h incubation with 1 μg·mL^−1^ of doxycycline, and proteins in the cells were detected using the indicated antibodies (*n* = 3). (C) After incubation of CDT1‐WT or CDT1‐NGB‐NDL expressing cells with 1 μg·mL^−1^ of doxycycline, 2 × 10^4^ cells were collected at 24, 48, and 72 h, and immunoblotting was performed to detect the indicated proteins in the collected cells (*n* = 3). (D) Soluble and chromatin fractions were obtained from CDT1‐WT or CDT1‐NGB‐NDL expressing cells after incubation for 0–72 h in the presence of 1 μg·mL^−1^ of doxycycline. Immunoblotting was performed to detect the indicated proteins in each fraction (*n* = 3).

CDT1 overexpression induces chromosomal damage and checkpoint activation (Fig. 5) [16, 19, 23, 35]. Herein, CDT1‐NGB‐NDL overexpression strongly inhibited cell proliferation (Fig. 5A). ATR was found to be promptly activated by autophosphorylation at Thr1989 in response to replication stress, and ATR‐dependent activation of CHK1 is the initial step of replication checkpoint pathways [36, 37]. We observed ATR and CHK1 phosphorylation 24 h after inducing the expression of CDT1‐NGB‐NDL (Fig. 5B), suggesting that inhibition of replication fork progression by CDT1‐NGB‐NDL induces activation of the ATR‐mediated checkpoint pathway. We also observed an increase in phosphorylated histone H2AX (γH2AX), a DSB marker, following CDT1‐NGB‐NDL induction (Fig. 5C). Moreover, MRE11, RAD51, and RPA2, which participate in homologous recombination and are known to be associated with stalled replication forks [38], accumulated in the chromatin fractions after the induction of CDT1‐NGB‐NDL expression (Fig. 5D). Taken together, these results suggest that CDT1‐mediated inhibition of replication fork progression evokes abnormalities of DNA structure that are resolved by homologous recombination.

## Discussion

In this study, we investigated the mechanism through which excess CDT1 inhibits the progression of replication forks in human cells. We found that overexpression of mutant CDT1 carrying decreased licensing activity induced cell cycle arrest in the S phase, suggesting that CDT1 inhibits replication fork progression independent of the induction of rereplication. This notion is consistent with our previous study, in which the mouse geminin fragment (79–130 amino acid region), which binds to CDT1 without inhibiting licensing activity, relieved replication repression by CDT1 in *Xenopus* egg extracts [24], implying that the licensing activity of CDT1 is not involved in the inhibitory effect on replication fork progression. This is also consistent with the findings of a recent report [31], where a mutation in the C‐terminal Mcm6‐binding domain of CDT1 was shown to play a role in inhibiting cell cycle progression, suggesting that the inhibitory effect of CDT1 requires interaction with Mcm6 through its C‐terminal domain. CDT1 reportedly stimulates DNA helicase activity in the purified Mcm4/6/7 complex. However, an abundance of CDT1 was associated with inhibition of DNA helicase activity [39]. Thus, CDT1 may bind to the MCM complex and inhibit its DNA helicase activity in S‐phase cells, thereby suppressing replication fork progression.

Flow cytometric analysis of the cell cycle profile revealed that the overexpression of wild‐type CDT1 did not increase the population of cells with DNA content ranging between 2C and 4C, which corresponds to the early to mid‐S phase (Fig. 2A). However, overexpression of the nondegradable form of CDT1, in which both the PIP‐box and Cy‐motif were mutated, resulted in the accumulation of cells with DNA content ranging between 2C and 4C (Fig. S1).

Although the expression of CDT1‐MC inhibited cell cycle progression, the C‐terminal fragment of CDT1 was insufficient to bind Mcm6 and inhibit cell cycle progression, suggesting that the middle region of CDT1 is pivotal to mediating these activities. The middle region of CDT1 was found to be essential for its licensing activity [5], although the actual function remains to be elucidated. Recently, we reported that *Xenopus* CDT1 self‐associates via its middle region. Mutations in two β‐strands, S1 and S2, of WHD in the middle region attenuated self‐association and largely decreased licensing activity [34]. We observed that the expression of CDT1‐NGB‐S1S2 did not increase the population of S‐phase cells, despite the observation that the expression levels of CDT1‐NGB‐S1S2 were comparable to those of CDT1‐NGB‐NDL. CDT1‐NGB‐S1S2 bound to Mcm6 to a similar extent as that of wild‐type CDT1, suggesting that the interaction between CDT1 and Mcm6 was insufficient to inhibit CMG helicase. Thus, in addition to the interaction with Mcm6, the self‐association of CDT1 and/or the uncharacterized properties of β‐sheet structures in the WHD in the middle region of CDT1 may be essential for inhibiting replication forks.

Our results indicate that overexpression of CDT1‐NGB‐NDL induced growth inhibition and activation of the ATR‐mediated checkpoint pathway. Moreover, phosphorylation of H2AX and chromatin association of MRE11, RPA, and RAD51 was also induced. These results suggest that arrested replication forks induced by CDT1‐NGB‐NDL eventually collapse and that homologous recombination participates in the repair process. Thus, ectopic activation of CDT1 could provoke serious replication stress by inducing rereplication and fork stalling, implying an association with the genome instability observed in numerous cancer cells. CDT1 was found to be overexpressed in several human cancer cells [14, 15, 16]. In addition, CDT1 has been identified as a putative oncogene, and its overexpression reportedly results in tumor formation *in vivo* [35, 40, 41, 42, 43]. Tumor cells overexpressing CDT1 show numerical and structural aberrations in chromosomes [41]. Moreover, high CDT1 expression was markedly associated with poor prognosis in patients with breast, hepatocellular, and gastric cancer [17, 18, 44]. Accordingly, replication stress induced by ectopic CDT1 may play a crucial role in tumor formation and progression. Ratnayeke *et al*. [31] reported that CDT1 is present along with the fired origin until its degradation and inhibits CMG helicase at replication forks to separate licensing from DNA synthesis. We propose that CDT1 contributes to the inhibition of CMG helicase and causes an increase in genome instability by stalling both replication and rereplication forks when CDT1 activity is deregulated.

Re‐replicating forks reportedly exhibit slower progression and limited elongation of the nascent DNA strand [45, 46], suggesting that replication fork progression is restricted by unknown mechanisms. Multiple rounds of rereplication have been suggested to cause head‐to‐tail collisions between replication forks, resulting in replication fork stalling [47]. Recently, RAD51, which binds to the stalled replication fork and protects it from degradation, was shown to physically impede the DNA template and hinder replication fork progression [48]. Based on our findings, we suggest that CDT1 inhibits replication fork progression by interacting with CMG helicase via WHD in its middle and C‐terminal regions. Our findings would contribute to the understanding of the mechanisms of tumorigenesis and tumor progression caused by replication stress induced by aberrant activation of licensing activity.

## Conflict of interest

The authors declare no conflict of interest.

## Author contributions

TT conceived and designed the study; ST supervised the study. TT, NT, RT, YA, YY, and YN conducted experiments and analyzed results. TT, YA, and ST wrote the manuscript. All authors reviewed and approved the manuscript.

## Supporting information


**Fig. S1.** Expression of non‐degradable form of CDT1 inhibits cell cycle progression at S phase. (A) Expression of non‐degradable CDT1 (CDT1‐ND) was induced by incubation of CDT1‐ND‐expressing cells with 1 μg·mL^−1^ of doxycycline for 48 h. (B) After 48‐h incubation with doxycycline, the cell cycle profiles of CDT1‐ND‐expressing cells were analyzed by flow cytometry.

## Data Availability

The data that support the findings of this study are available from the corresponding author [s.tada@phar.toho-u.ac.jp] upon reasonable request.
