# Supplementary material for: Overexpression of CDT1 inhibits cell cycle progression at S phase by interacting with the mini‐chromosome maintenance complex and causes DNA damage
Source: FEBS Open Bio. 2025 Sep 26;16(2):352–64. doi: 10.1002/2211-5463.70127 (PMC12871567; doi:10.1002/2211-5463.70127)
Supplement: Supplementary file 1 — Fig. S1. Expression of non‐degradable form of CDT1 inhibits cell cycle progression at S phase. (A) Expression of non‐degradable CDT1 (CDT1‐ND) was induced by incubation of CDT1‐ND‐expressing cells with 1 μg·mL−1 of doxycycline for 48 h. (B) After 48‐h incubation with doxycycline, the cell cycle profiles of CDT1‐ND‐expressing cells were analyzed by flow cytometry. [file FEB4-16-352-s001.docx]

**Supporting Information**


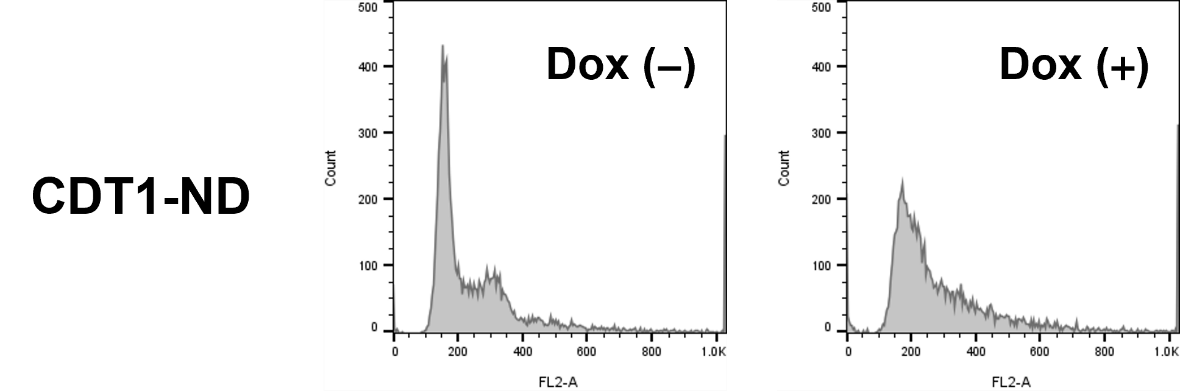


**A**

**B**

2C

4C

2C

4C


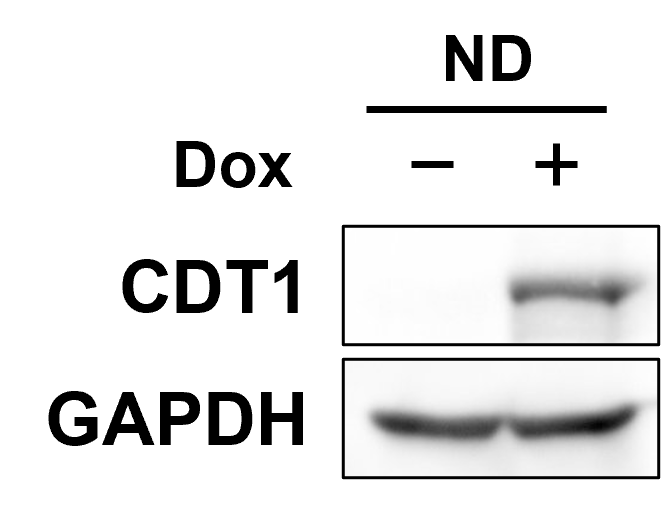


**Fig. S1 Expression of non-degradable form of CDT1 inhibits cell cycle progression at S phase** (A) Expression of non-degradable CDT1 (CDT1-ND) was induced by incubation of CDT1-ND-expressing cells with 1 μg/mL of doxycycline for 48 h. (B) After 48-h incubation with doxycycline, the cell cycle profiles of CDT1-ND-expressing cells were analyzed by flow cytometry.
